# Supplementary material for: Venomix: a simple bioinformatic pipeline for identifying and characterizing toxin gene candidates from transcriptomic data
Source: PeerJ. 2018 Jul 31;6:e5361. doi: 10.7717/peerj.5361 (PMC6074769; doi:10.7717/peerj.5361)
Supplement: Supplemental Information 3 [file peerj-06-5361-s003.gz › FinalOutput_E-6/Venom_allergen_5_1/finaltree.pdf]

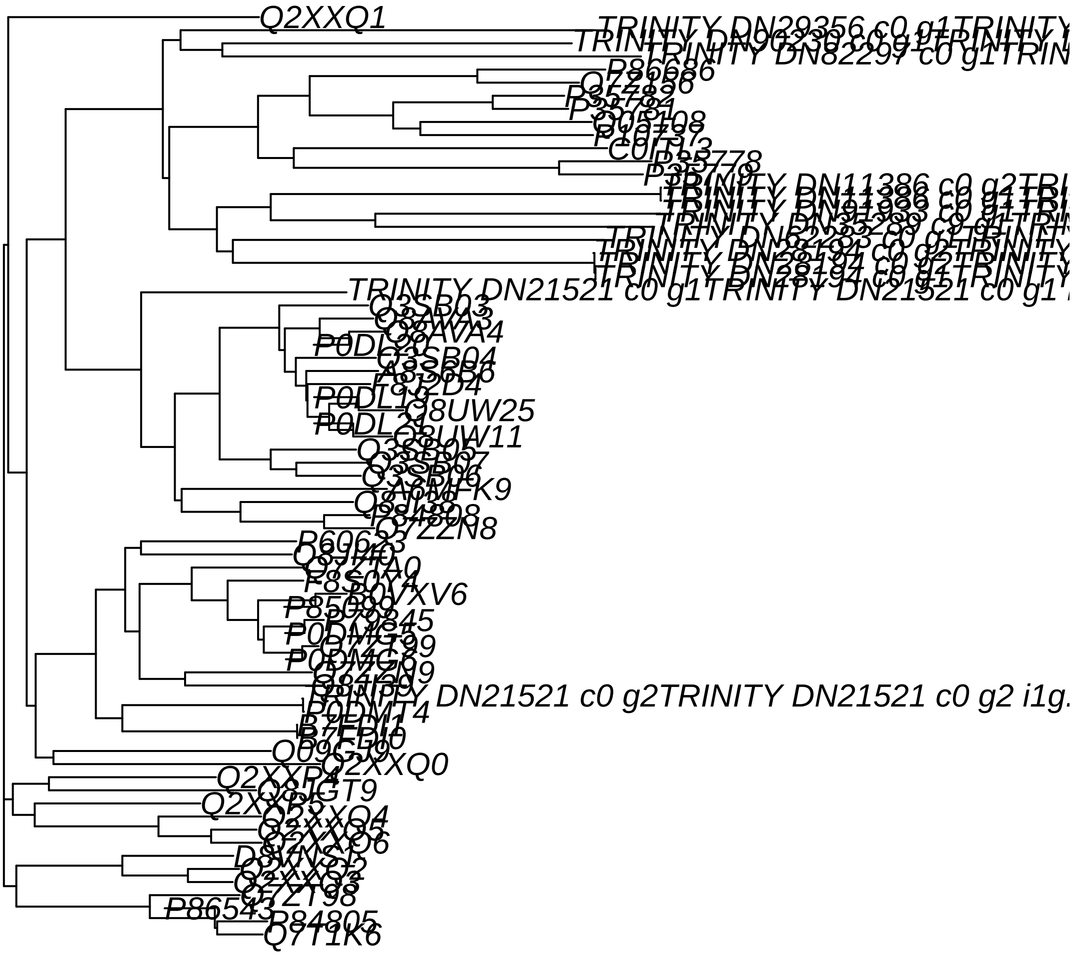
Q2XXQ1
TRINITY DN29356 c0 g1TRINITY
P86686
P35778
TRINITY DN21521 c0 g1TRINITY DN21521 c0 g1
Q3SB03
Q6AVA4
P0DL20
Q3SB04
Q8UW25
P0DL21
Q8UW11
A0MFK9
Q7ZZN8
P60623
Q7ZTA0
P0DMT4
Q2XXQ0
Q8JGT9
Q2XXQ4
Q2XXQ6
Q7ZT98
P86543
P84805
Q7T1K6
TRINITY DN21521 c0 g2TRINITY DN21521 c0 g2 i1g.
